# Functional graph contrastive learning of hyperscanning EEG reveals emotional contagion evoked by stereotype-based stressors

**DOI:** 10.3389/fnhum.2023.1298845

**Published:** 2023-11-22

**Authors:** Jingyun Huang, Rachel C. Amey, Mengting Liu, Chad E. Forbes

**Affiliations:** ^1^School of Computer Science and Engineering, Sun Yat-sen University, Guangzhou, China; ^2^Army Research Institute for the Behavioral and Social Sciences, Fort Belvoir, VA, United States; ^3^School of Biomedical Engineering, Sun Yat-sen University, Shenzhen, China; ^4^Department of Psychology, Florida Atlantic University, Boca Raton, FL, United States

**Keywords:** emotional contagion, graph contrastive learning, graph representation learning, stereotype-based stressor, graph classification

## Abstract

**Introduction:**

This study delves into the intricacies of emotional contagion and its impact on performance within dyadic interactions. Specifically, it focuses on the context of stereotype-based stress (SBS) during collaborative problem-solving tasks among female pairs. Through an exploration of emotional contagion, this study seeks to unveil its underlying mechanisms and effects.

**Methods:**

Leveraging EEG-based hyperscanning technology, we introduced an innovative approach known as the functional graph contrastive learning (fGCL), which extracts subject-invariant representations of neural activity patterns from feedback trials. These representations are further subjected to analysis using the dynamic graph classification (DGC) model, aimed at dissecting the process of emotional contagion along three independent temporal stages.

**Results:**

The results underscore the substantial role of emotional contagion in shaping the trajectories of participants' performance during collaborative tasks in the presence of SBS conditions.

**Discussion:**

Overall, our research contributes invaluable insights into the neural underpinnings of emotional contagion, thereby enriching our comprehension of the complexities underlying social interactions and emotional dynamics.

## 1 Introduction

Emotional contagion refers to the sharing of emotional states between individuals, and it has been observed in both animal and human models that the infectivity of negative emotions is much greater than that of positive emotions (Goldenberg and Gross, 2020). Negative emotional contagion has a powerful effect on our relationships—family, friends, teams, etc.—and can lead, for example, to depressive behavior in healthy people who live with depressed individuals. It is urgent to understand the mechanism of emotional contagion, especially negative emotional contagion. At present, the emotional contagion models mostly adopt behavioral analysis and questionnaires, which are often affected by subjects' subjective factors. They have mainly focused on behavioral experiment such as analyzing people's posts containing emotional information to extract affective evidence (Kramer et al., 2014), using the Positive And Negative Affective Schedule (PANAS) scale to measure positive and negative emotions as a quantitive research (Alhubaishy and Benedicenti, 2017) and the mathematical simulation model of emotional contagion in crowd evacuation (Zhang et al., 2020).

Although behavioral analysis and questionnaires can provide valuable insights into emotional contagion, they have limitations in terms of capturing the neural mechanisms, timing, and subtleties of this phenomenon. To overcome these limitations, researchers have turned to EEG-based hyperscanning, a technology that records electroencephalographic (EEG) data from multiple participants simultaneously. This approach complements traditional behavioral analysis and questionnaires by providing a more direct and precise real-time examination of the underlying brain activities associated with emotional contagion. EEG-based hyperscanning technology has proven effective in capturing brain states during affective communication. For instance, when an individual experiences specific emotions such as sadness, joy, or fear, their brain activity may influence the brain activity of others they interact with, thus bearing implications for emotional contagion (Liu et al., 2018).

However, the significant inter-subject variability of emotion-related EEG signals poses a great challenge for cross-individual emotional representation extraction (Shen et al., 2022). In most cases, the accuracy of the intra-subject emotion classification is higher than that of inter-subject classification with the same classifier (Li et al., 2020; Suhaimi et al., 2020). This limitation in the generalization of emotion classifiers may be attributed to individual differences in EEG-based emotional representations, influenced by factors such as personality, dispositional affect, and genotype (Hamann and Canli, 2004). Furthermore, individual variance in patterns of brain connectivity reveals that the inter-subject contrast plays a significant role in cognitive analysis (Finn et al., 2015). These previous findings suggest that EEG emotional representation should not be extracted without considering the individual difference.

In this study, we investigated whether women who experienced social identity threat could transmit their stress to women who were not under threat, using a process known as stereotype-based stress (SBS) contagion and examined how this collective stress affected women in a dyadic performance context. Previous research has shown that SBS contexts typically engender a variety of behavioral and physiological SBS responses including sustained vACC activation, unique neural network configurations, and enhanced connectivity between regions integral for emotion (dACC, vACC, and mPFC) and saliency networks (IPL, insula, and STS). This evidence collectively suggests increased emotional processing and heightened awareness of negatively arousing or stressful information (Forbes et al., 2018; Liu et al., 2020, 2021; Amey et al., 2022). We sought to understand if threatened women can transmit their stress to otherwise non-threatened partners, does it hurt or benefit the woman directly under threat, and to what extent can this come at a cost to their otherwise non-threatened partners? To this end, we designed an experimental paradigm of emotional contagion by using discussion and learning scenarios, and try to understand the neurological mechanism of emotional contagion by using EEG-based hyperscanning technology combined with a data-driven approach called functional graph contrastive learning (fGCL). This method extracts the subject-invariant emotional representations while preserving functional connectivity (FC) information. Subsequently, we conducted a downstream analysis to infer and explore the process of emotional contagion. The formulation of fGCL is grounded on the assumption that the neural activities of the subjects are in a similar state when they receive the same segment of emotional stimuli (i.e., the displayed CORRECT or WRONG responses on the screen). Based on this fundamental idea, we aim to learn subject-invariant representations of EEG signals in the embedding space underlying similar mental activities. Specifically, fGCL mainly consists of two components, i.e., the spectral-based graph convolutional network (spectral GCN) encoder and a two-layer multi-layer perceptron (MLP). It maximizes the similarities of the representations in response to identical emotional stimuli while minimizing the similarities between signals corresponding to different stimuli. In the downstream analysis, we employ a classifier known as dynamic graph classification (DGC), which utilizes the graph embeddings extracted by the trained fGCL encoder as input to identify emotional stimuli type (i.e., *CORRECT* or *WRONG* responses) during emotional contagion within dyads. These representations, extracted based on semantically meaningful settings, are expected to be informative and generalizable for the downstream analysis.

The method presents three essential characteristics:

- The presented model can extract EEG signal representations with inter-individual commonality and remove the individual differences, which more effectively summarize the internal neural activity pattern.
- A deep learning model that is more effective than traditional statistical analysis and behavioral analysis methods to investigate the emotional contagion mechanism.
- The graph data structure-based analysis is more aligned with functional brain network structure and thus yield more intuitive and informative results.

## 2 Method

### 2.1 Graph construction and analysis procedure

As individual difference exists in inter-subject functional connectivity (FC), a statistical dependency quantifies the connection strengths between brain region of interests (ROIs) (Mueller et al., 2013), and our aim is to preserve the FC information for more effective emotional analysis. To this end, we adopt graphs, which are naturally suitable for modeling brain topology. In this approach, we project ROIs onto the nodes of a graph and the weighted edges connect nodes; this overcomes the limitation of traditional 2D grid-like structure, where models might fail to explore and exploit the complex FC (Demir et al., 2021).

Given a dataset ${\left\{\left({𝒢}_{i}^{j},y_{i}\right)\right\}}_{i=1}^{N}$ with N individuals, where *y*_*i*_ = {0, 1} represents the label of *i*-th graph; ${𝒢}_{i}^{j}=\left\{{𝒱}_{i}^{j},{ℰ}_{i}^{j}\right\}$ corresponds to the *j*-th view within augmented views (see Section 3.7), where Pearson correlations among ROIs are calculated as node features; $X_{i}^{j}\in {ℛ}^{ROIs\times ROIs}$, where *ROIs* denotes the number of ROI in a graph; and $x_{n}=X{\left[n,:\right]}^{T}$ is the ROIs-dimensional node feature for node $v_{n}\in 𝒱$, containing FC information. Partial correlations between ROIs is used as edge features ${ℋ}_{i}^{j}\in {ℛ}^{ROIs\times ROIs}$, where $h_{nn^{\prime }}=ℋ{\left[n,n^{\prime }\right]}^{T}$ is the edge feature of edge $e_{nn^{\prime }}\in ℰ$.

**Figure 1:**
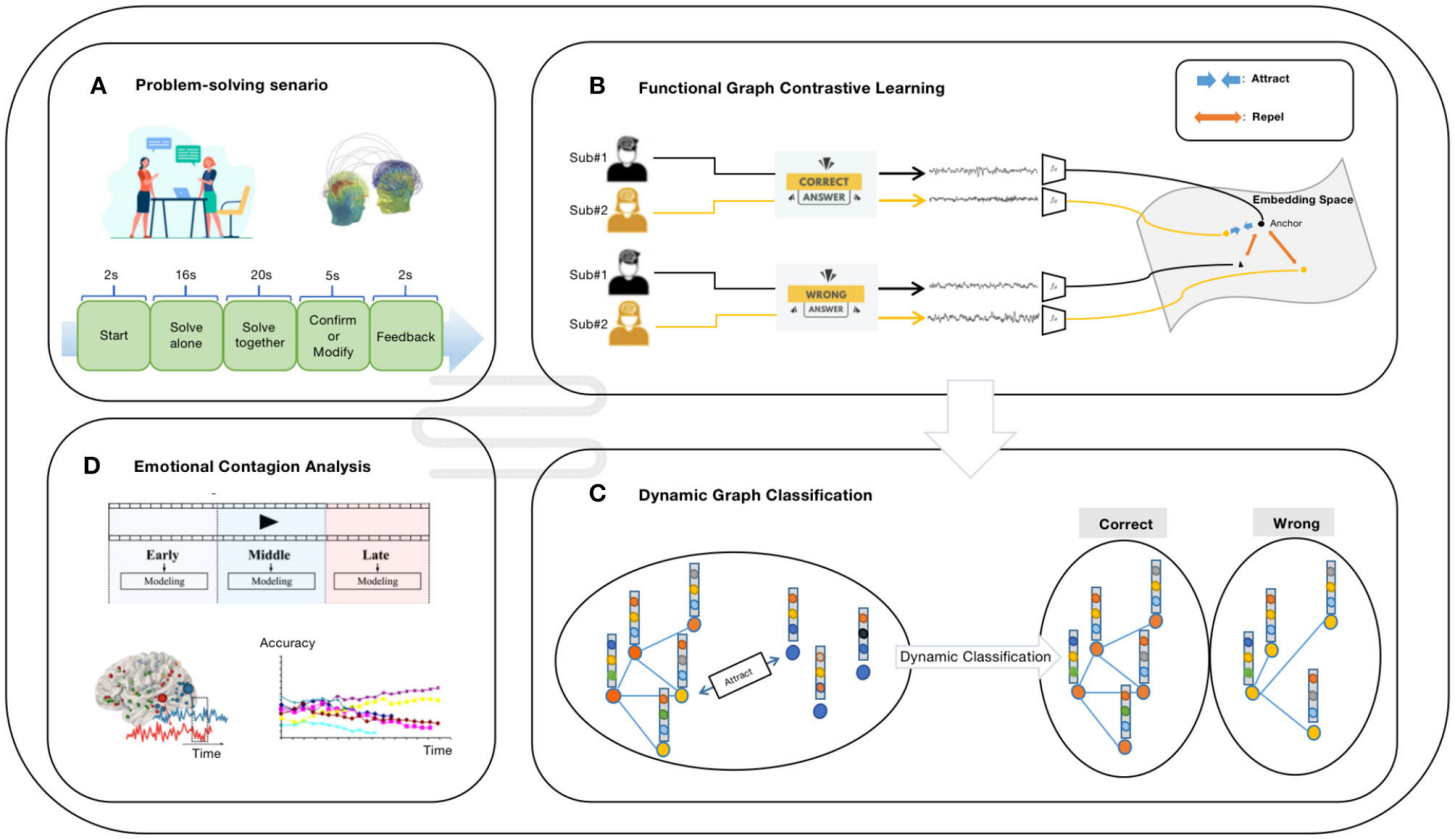
The schematic overview of our study. **(A)** The experiment setting. Subjects in one dyad answer mathematical questions while recording their EEG signals simultaneously. Each participant need to solve mathematical questions both individually and together with their partners before confirming the final decision and receiving the feedback from the computer screen. **(B)** The illustration of functional graph contrastive learning. Given a minibatch of graph data representing subjects in the same dyad, the fGCL encoder is able to extract subject-invariant graph embeddings. **(C)** The illustration of dynamic graph classification. The DGC classifier dynamically updates the population graph, which is constituted of graph embeddings as isolated nodes initially and predicts the label of each node ultimately. **(D)** The downstream analysis. The dataset is divided into early stage, middle stage, and late stage according to the scenario of tasks to perform emotional contagion analysis using the trained DGC classifier.

As illustrated in Figure 1, the functional graph contrastive learning (fGCL) encoder learns to extract embeddings of each graph as subject-invariant representations (i.e., maximizing the representational similarity of EEG signals belonging to the similar scenario while minimizing it for dissimilar ones); by leveraging this encoder, we construct a population graph where a collection of subject-invariant embeddings of EEG signals represent as nodes. Given this, the dynamic graph classification (DGC) classifier is trained to perform classification task on three stages (early, middle, and late). The results can be further utilized for emotional contagion analysis (see Section 4.3).

#### 2.1.1 The functional Graph Contrastive Learning encoder

**Figure 2:**
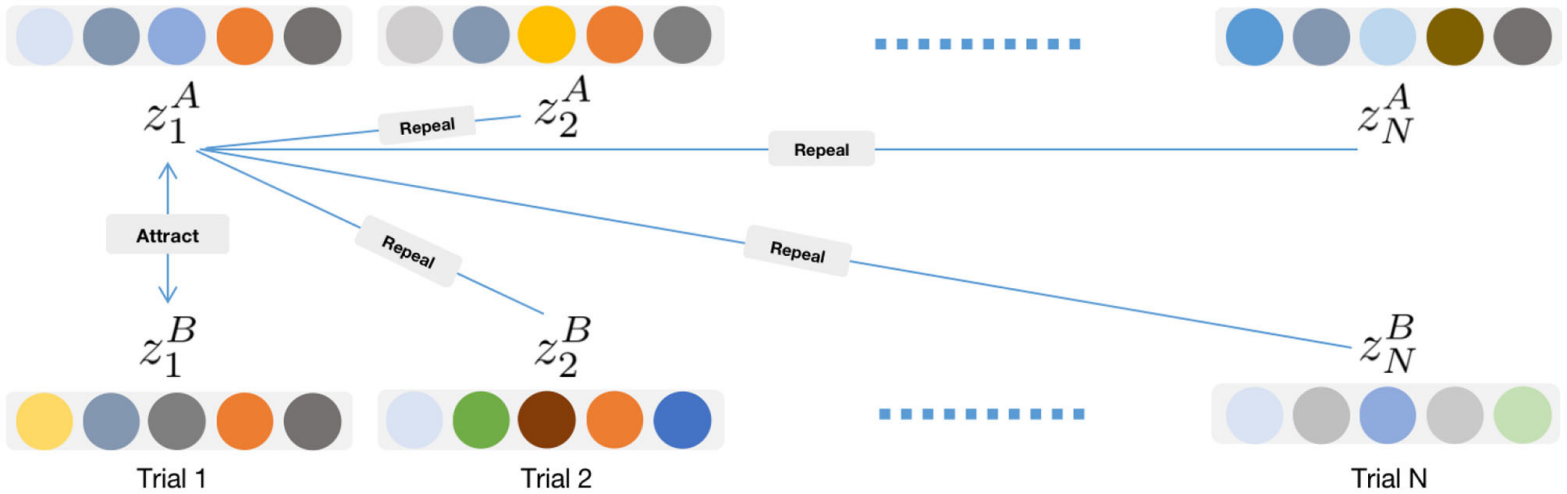
The illustration of the construction of positive pair and negative pair in graph contrastive learning. In a minibatch, the embedding $z_{1}^{A}$ as an anchor forms a positive pair with $z_{1}^{B}$, and all other embeddings form negative pairs with $z_{1}^{A}$.

The fGCL encoder *f* takes N-paired graphs (minibatch) $\left\{{𝒢}_{i}^{A}|i=1,..,N\right\}$ and $\left\{{𝒢}_{j}^{B}|j=1,..,N\right\}$ as inputs to generate subject-invariant embeddings $\left\{z_{i}^{A}|i=1,..,N\right\}$ and $\left\{z_{j}^{B}|j=1,..,N\right\}$ of graphs for each type of trial (correct or wrong feedback in our experiment). It adopts spectral graph convolutional neural network consisting of two Chebshev spectral graph convolutional layers (Defferrard et al., 2016), each followed by a TopK pooling layer (Cangea et al., 2018) as model backbone to capture representations and retain important nodes in the iterative aggregation. Additionally, global average pooling layer and global mean pooling layer are used to capture the global information. Finally, a two-layer multi-layer perceptron (MLP) is employed to output the graph embeddings. Similar to SimCLR framework (Chen et al., 2020) with InfoNCE loss, we adopt the contrastive loss function for the anchor embedding $z_{i}^{A}$ defined as:


(1) $$\begin{array}{l} \;L(z_{i}^{A})=\\ -log(\frac{exp(sim(z_{i}^{A},z_{i}^{B})/\tau )}{\sum_{j=1}^{N}I_{j\neq i}exp(sim(z_{i}^{A},z_{j}^{A})/\tau )+\sum_{j=1}^{N}exp(sim(z_{i}^{A},z_{j}^{B})/\tau )}) \end{array}$$

where *I*_*j*≠*i*_ = {0, 1} is an indicator, which is set to 1 when *j*≠*i*, and τ is a temperature factor to adjust the attractiveness strength. Overall, this loss function increases the attractiveness of positive pair ($z_{i}^{A}$ and $z_{i}^{B}$) and decrease the attractiveness of negative pair ($z_{i}^{A}$ and others) in the embeddding space (Figure 2). The similarity between two embeddings is computed by


(2) $$\begin{array}{l} sim\left(z_{i}^{A},z_{j}^{B}\right)=\frac{z_{i}^{A}\cdot z_{j}^{B}}{\left||z_{i}^{A}||\;||z_{j}^{B}|\right|} \end{array}$$

Eventually, the accumulated loss of the minibatch is computed by


(3) $$\begin{array}{l} L=\overset{N}{\underset{i=1}{\sum }}L\left(z_{i}^{A}\right)+\overset{N}{\underset{i=1}{\sum }}L\left(z_{i}^{B}\right) \end{array}$$

The spectral convolution blocks of fGCL comprise the Chebyshev spectral graph convolutional operator (i.e., ChebConv layer), which is defined by:


(4) $$\begin{array}{l} X^{\prime }=\overset{K}{\underset{k=1}{\sum }}{Ƶ}^{\left(k\right)}\left(X\right)\cdot \theta^{\left(k\right)} \end{array}$$

where *K* is the Chebyshev filter size, ${𝒵}^{\left(k\right)}\left(X\right)$ is computed recursively by ${𝒵}^{\left(1\right)}=X$, ${𝒵}^{\left(2\right)}=\tilde{L}\cdot X$, all the way to ${𝒵}^{\left(k\right)}=2\cdot \tilde{L}\cdot {𝒵}^{\left(k-1\right)}-{𝒵}^{\left(k-2\right)}$, and $\tilde{L}$ denotes the scaled and normalized Laplacian $\frac{2L}{\lambda_{max}}-I$, where λ_*max*_ is the largest eigenvalue of *L*. $\theta^{\left(k\right)}$ are learnable parameters. To prevent overfitting, each ChebConv layer is followed by a TopK pooling layer, which downsamples graphs and reduces their dimensionality while retaining the most relevant nodes (i.e., top *K* nodes), leading the model to focus on meaningful information. The resulting embeddings of graphs, optimized through graph contrastive learning, are then fed into the downstream classifier for graph classification tasks.

#### 2.1.2 Downstream analysis on the outcomes of emotional contagion

After the graph contrastive learning phase, individual differences have been eliminated in the embedding space. This means that the extracted representations preserve the commonality of neural activity patterns in response to both correct and wrong feedbacks. To fully utilize these aligned representations, we adopt the dynamic graph classification (DGC) model to perform emotional contagion state analysis.

Initially, the DGC takes a population graph with isolated nodes as the input. By iteratively projecting node features onto a new feature space, it is able to construct new edges based on the top-K connection strengths defined as:


(5) $$\begin{array}{l} v_{i}^{P^{\prime }}=\underset{j\in {ℰ}_{\left(i,\cdot \right)}^{P}}{\sum }h_{\text{Θ}}\left(v_{i}^{P}||v_{j}^{P}-v_{i}^{P}\right),\;{ℰ}^{P}=\text{KNN}{\left({𝒱}^{P}\right)}_{\text{topK}} \end{array}$$

where || is the concatenation function and *h*_Θ_ denotes a fully connected neural network with a set of learnable parameters Θ. Initially, *h*_Θ_ projects the node features $v_{i}^{P}$ and $v_{i}^{P}-v_{j}^{P}$ to obtain the edge features *e*_*ij*_, and the summation of these edge features is used to update the node features. This process takes into account both global information captured by $v_{i}^{P}$ and local neighborhood information captured by $v_{j}^{P}-v_{i}^{P}$. Edges are constructed by selecting the top-K closest nodes through pairwise distance matrix computations in the feature space for each individual node. During each dynamic graph update, connections between closer nodes are strengthened, while edges with more distant nodes are deprecated. The final layer of the DGC is a fully connected neural network used to predict the class of each node.

After the training procedure, the DCG have learned the pattern of the neural response to the correct and wrong feedbacks. The classification tasks are conducted based on the early, middle, and late stages of the problem-solving tasks. When individuals encounter the wrong feedback, there is stronger activation in brain regions associated with emotions, such as the vACC, dACC, and mPFC. In the case of DMT-PST dyads, when DMT actors transmit negative emotions to DMT partners and their own negative emotions dissipate, the brain exhibits more distinct response patterns for positive and negative feedbacks. This makes it easier for the classifier to correctly classify the feedback types, reflecting an improvement in classification performance. However, for DMT partners, due to the influence of negative emotions, brain activity becomes more complex, making it difficult for the model to distinguish the correct patterns. As for the PST-PST dyads, there are no such phenomena, indicating that there is no fluctuation in classification performance over time.

The algorithm of training the fGCL encoder and the downstream classifier DGC are summarized in Algorithm 1.

**Algorithm 1:**
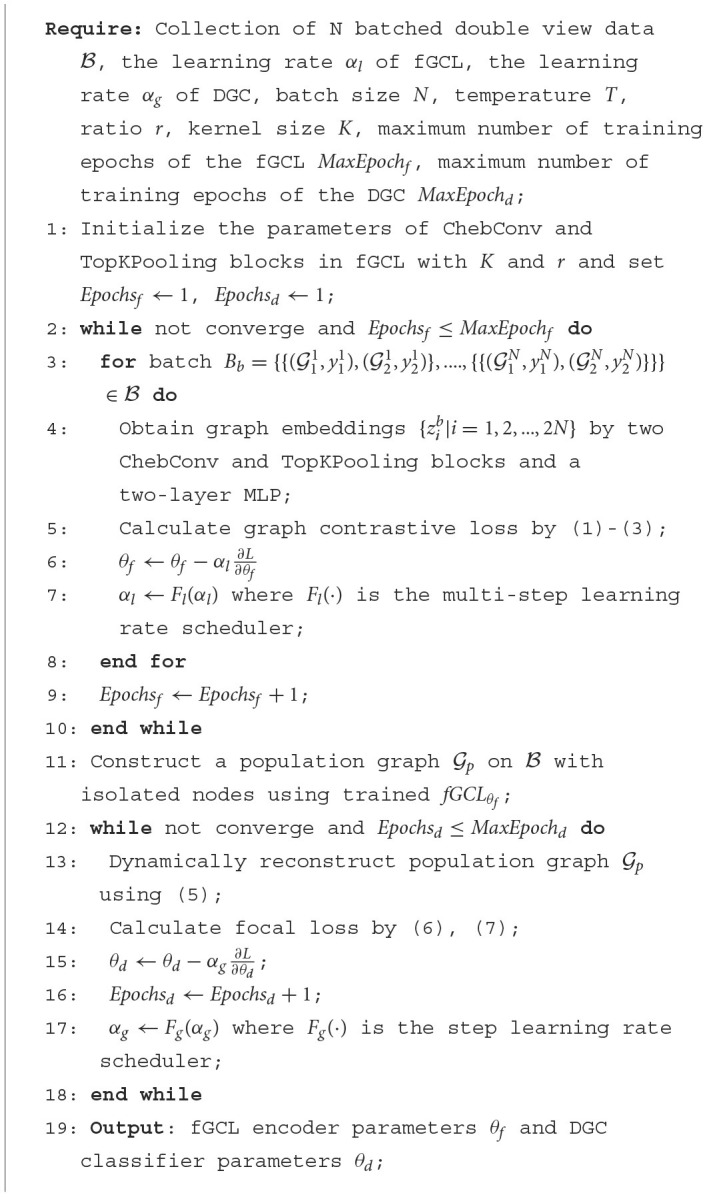
The training algorithm of fGCL+DGC — learning graph embedding and classification.

## 3 Experiment

### 3.1 Participants

Eighteen white female students who granted written consent participated in the study for payment. Participants were recruited for the study if they expressed knowledge of the stereotype that men are better at math than women. Specifically, all participants responded with a three or lower to the following question during a pre-study screening: “Regardless of what you think, what is the stereotype that people have about women's and men's math ability” (1 = Men are better than women; 7 = Women are better than men). Participants were paired into nine dyads.

### 3.2 Procedure

Upon arrival to the lab, partners of each dyad met for the first time while signing consent forms; they were then prepared for EEG recording. Each member of the dyad was seated in their own soundproof chamber in front of a computer screen and iPad tablet. Dyads were randomly assigned to either an SBS/diagnostic math test condition (DMT, *n* = 7 dyads) or a control/problem-solving task condition (PST, *n* = 2 dyads). In the DMT condition, one participant (referred to as the “actor”) was exposed to SBS by being told they would complete tasks that were diagnostic of their math intelligence. They also completed demographic questions that included a gender query, had pre-recorded instructions read aloud to them in a male voice through headphones, and were prepped for EEG recording by at least one male experimenter. In contrast, the DMT actor's interaction partner (referred to as the “partner”) and all participants in the PST condition were informed that they would be completing tasks that would inform researchers about the different types of problem-solving techniques they prefer (Forbes and Leitner, 2014; Forbes et al., 2015), completed demographic questions that excluded the gender query, had prerecorded instructions read aloud to them by a female voice through headphones, and were set up by female experimenters. Thus, DMT partners and both participants in the PST condition were always placed in stereotype neutral/stress-free contexts. That is, only the condition of the actor varied across dyad conditions. After an initial set of instructions, participants were connected via webcam on their iPad tablet to facilitate face-to-face communication during the interactive math task (described in Section 3.3). Participants were able to see one another through the duration of the interactive math task. When the interactive math task was completed, participants answered a series of questionnaires alone (iPads were removed from the EEG chambers), were debriefed, and were compensated for their participation with cash or course credit.

### 3.3 Interactive math task

Actors and partners simultaneously completed a 100-problem math task consisting of standard multiplication and division problems (e.g., 10 × 20=) that they solved both alone and together. Initial pilot tests confirmed that the problems selected varied in degree of difficulty (easy, medium, and hard), ensuring all participants would solve problems correctly and incorrectly, thus exposing them to both positive and negative performance feedback. Actors and partners were first presented with the same math problem to solve alone for 16 s. During this solo time, participants were given three answer choices below each problem (A, B, or C), with the answer to each problem randomly presented in one of the three answer positions. Participants mentally completed all problems without scratch paper and made all answer selections via a button box placed in their laps. This solo answer was used for all performance outcomes in our analyses. After participants entered their solo answer, they were prompted with a screen that said, “Please discuss the answer to the problem with your partner.” At this time, participants were given 20 s to discuss their answers with their partners. Participants were then given 5 s to change or confirm their answer to the math problem they just solved alone. After submitting their final response, participants received feedback for 2 s that indicated whether their final answer was correct or incorrect (presented as the words *CORRECT* or *WRONG* written in black on a white screen).

### 3.4 EEG recording

Consistent with Forbes's research (Forbes et al., 2018), continuous EEG activity was recorded from each member of the dyad using an ActiveTwo head cap and the ActiveTwo Biosemi system (BioSemi, Amsterdam, Netherlands). Recordings were collected from 64 Ag-AgCl scalp electrodes and from bilateral mastoids. Two electrodes were placed next to each other 1 cm below the right eye to record eye-blink responses. A ground electrode was established by BioSemi's common Mode Sense active electrode and Driven Right Leg passive electrode. EEG activity was digitized with ActiView software (BioSemi) and sampled at 2,048 Hz. Data were downsampled post-acquisition and analyzed at 512 Hz. In our data-driven analysis, we only consider the feedback trials (i.e., *CORRECT* or *WRONG* responses) to better capture elicited emotional patterns when participants are evoked by stimuli.

### 3.5 Data preprocessing

For feedback analyses, the EEG signal was epoched and stimulus-locked from 500 ms pre-feedback presentation to 2,000 ms post-feedback presentation. EEG artifacts were removed via FASTER Fully Automated Statistical Thresholding for EEG artifact Rejection (FASTER) (Nolan et al., 2010), an automated approach to cleaning EEG data that is based on multiple iterations of independent component and statistical thresholding analyses. Specifically, raw EEG data were initially filtered through a band-pass FIR filter between 0.3 and 55 Hz. The EEG channels with significant unusual variance (absolute *z* score larger than three standard deviations from the average), mean correlations with other channels, and Hurst exponents were removed and interpolated from neighboring electrodes using a spherical spline interpolation function. EEG signals were then epoched and baseline corrected. Epochs with significant unusual amplitude range, variance, and channel deviation were removed. The remaining epochs were then transformed through ICA. Independent components with significant unusual correlations with EOG channels, spatial kurtosis, slope in the filter band, Hurst exponent, and median gradient were subtracted and the EEG signal was reconstructed using the remaining independent components. Finally, EEG channels within single epochs with significant unusual variance, median gradient, amplitude range, and channel deviation were removed and interpolated from neighboring electrodes within the same epochs.

### 3.6 Source reconstruction

All a priori sources used in network connectivity analyses were identified and calculated via forward and inverse models utilized by MNE-python (Gramfort et al., 2013, 2014). The forward model solutions for all source locations located on the cortical sheet were computed using a three-layered boundary element model (Hämäläinen and Sarvas, 1989), constrained by the default average template of anatomical MNI MRI. Cortical surfaces extracted with FreeSurfer were sub-sampled to ~10,240 equally spaced vertices on each hemisphere. The noise covariance matrix for each individual was estimated from the pre-stimulus EEG recordings after preprocessing. The forward solution, noise covariance, and source covariance matrices were used to calculate the dynamic statistical parametric mapping (dSPM) estimated inverse operator (Dale et al., 1999, 2000). The inverse computation was done using a loose orientation constraint (loose = 0.2, depth = 0.8) (Lin et al., 2006). The dSPM inverse operators have been reported to help characterize distortions in cortical and subcortical regions and improve the bias accuracy of neural generators in deeper structures, e.g., the insula (Attal and Schwartz, 2013), by using depth weighting and a noise normalization approach. The cortical surface was divided into 68 anatomical regions (i.e., sources) of interest (ROIs; 34 in each hemisphere) based on the Desikan—Killiany atlas (Desikan et al., 2006), and signal within a seed voxel of each region was used to calculate the power within sources and phase locking (connectivity) between sources.

### 3.7 Implementation details

After the data preprocessing, 18 female individuals formed nine pairs, consisting of seven DMT-PST dyads and two PST-PST dyads. We then applied a sliding window technique to all basic views (the initial multi-ROI EEG time series without augmentation), denoted as 𝒳, which has 768 time points and 68 ROIs. The width and step size of the sliding window were set to 300 and 50, respectively, resulting in 10 augmented views (68 * 300) for each basic view (68 * 768). This augmentation increased the size of our dataset to 17,650 graphs, representing subjects who received either *WRONG* or *CORRECT* feedback. This augmentation allows the model to learn more general patterns. The dataset was split into training, testing, and validation sets in a 7:2:1 ratio.

In the graph contrastive learning procedure, within a N-sized minibatch, given a collection of graphs $\left\{{𝒢}_{i}^{j}|j=1,2,3,...,K\right\}$ representing i-th mathematical question, where *j* is an index denoting augmented views, *i* = 1, 2, ...., *N*. We enumerate all possible pairs within these graphs to form positive pairs. Within a dyad *D*_*m*_ consisting of subject $D_{m}^{A}$ and $D_{m}^{B}$, the K augmented views $\left\{{𝒢}_{i}^{j}|j=1,2,3,...,K\right\}$ of one of the members (e.g., $D_{m}^{A}$) represent the same mathematical question and exhibit similar neural activity patterns. Consequently, given one view ${𝒢}_{i}^{j}$ among these K augmented views, it forms positive pairs with the other views within the set, denoted as $\left\{{𝒢}_{i}^{k}|k=1,2,3,...,K,k\neq j\right\}$, and negative pairs with views representing other mathematical questions (i.e., $\left\{{𝒢}_{r}^{n}|n=1,2,3,...,K,r\neq i\right\}$). Additionally, when two subjects encounter the same mathematical question, one augmented view of subject $D_{m}^{A}$ in the m-th dyad should form positive pairs with the other K augmented views of subject $D_{m}^{B}$ in the same dyad. This process results in a total of 2K * (2K − 1) / 2 positive pairs (190 in our experiments). We trained the model for 700 epochs with early stopping and adopted a multi-step learning rate scheduler and Adam optimizer. The initial learning rate, weight decay, temperature, and batch size was empirically set to 0.001, 0.02, 0.5, and 68, respectively. The filter size in the Chebyshev spectral graph convolutional operator was set to 4, and the ratio of the TopK pooling layer was set to 0.5.

After obtaining the encoder *f*_θ_, graph features can be extracted as an embedding $z_{i}=f_{\theta }\left({𝒢}_{i}\right)\in R^{d}$ in the *d*-dimensional embedding space forming isolated nodes in the population graph initially. In the feedback type classification procedure, we use the basic view dataset (without sliding window augmentation) to train the DGC classifier for 100 epochs. We adopted focal loss, Equations (6) and (7), for the classifier to mitigate the aftermath of imbalanced data. We set α_*t*_ and γ to 0.5 and 2 empirically.


(6) $$\begin{array}{l} FL\left(p_{t}\right)=-\alpha_{t}{\left(1-p_{t}\right)}^{\gamma }log\left(p_{t}\right) \end{array}$$

(7) $$p_{t}=\left\{ \begin{array}{l} p\text{ if }y=1\\ 1-p\text{ otherwise}, \end{array}\right.$$

All methods are implemented with Pytorch and trained with GPU NVIDIA_GeForce_RTX_3090.

## 4 Experimental result

### 4.1 Graph embedding effectiveness analysis

**Figure 3:**
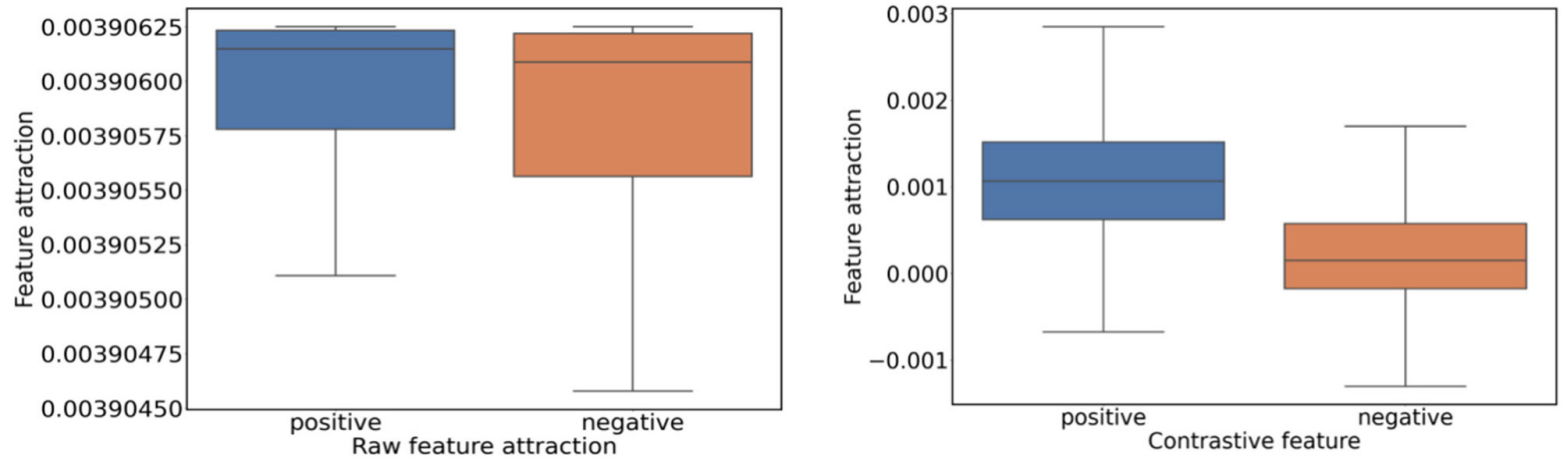
The feature attraction of graph raw features and contrastive features.

**Figure 4:**
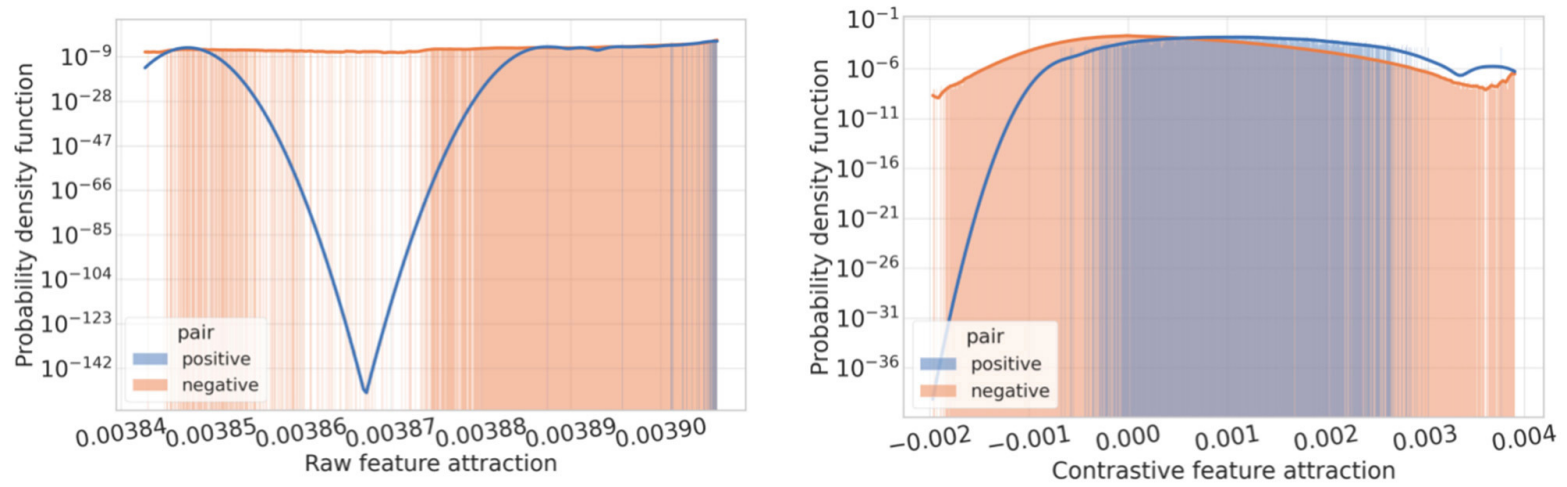
The probability density function of attractions on graph raw features and contrastive features.

We visualize the feature attraction of graph embeddings and raw features in the dataset. Figures 3, 4 indicate that the positive pairs have higher feature attraction than that of negative pairs. In addition, the feature attraction of negative pairs is close to 0, which verifies the effectiveness of fGCL in reducing the attraction of negative pairs and heightening the attraction of positive pairs. Conversely, the feature attraction on raw features display no significant differences, indicating identical similarity between positive and negative pairs.

### 4.2 Impact of emotional contagion on performance over time

Initial examinations of performance revealed a condition by time interaction, $Wald_{\chi }^{2}\left(2\right)$ = 13.72, *p* = 0.001. Specifically, DMT partner's performance decreased over time (*B* = −0.005 (SE = 0.002); $Wald_{\chi }^{2}\left(1\right)$ = 7.45, (95% Wald LL CI = −0.009; UL CI = −0.002), *p* = 0.006); for every one unit increase in time (trial number), the log odds of getting the question correct decreased by 0.005 units. In contrast, DMT actors and PST dyad members exhibited no over-time changes. Their performance remained stable (*p*'s > 0.11). Moreover, simple contrasts between conditions revealed performance differences between DMT actors and partners over the course of the task. At the beginning of the task, DMT partners had a higher probability of getting a question correct in comparison to the DMT actor (*p* = 0.002) and PST dyad members (*p* = 0.034). At the end of the task, DMT partners had a lower probability of getting a question correct in comparison to the partner (*p* = 0.004) and were largely comparable to PST dyad members (*p* = 0.089). DMT actors did not differ from PST dyad members (*p* = 0.35). Thus, at the end of the task, DMT partners underperformed in comparison to the actors. These findings support the possibility that DMT actors benefited from dyadic interactions at the expense of their partners.

### 4.3 Contagion stage analysis

In the analysis of the contagion stage, we divided the basic view dataset into training, testing, and validation sets in a 7:2:1 proportion. Subsequently, we extracted subject-invariant embeddings using the trained fGCL encoder for the classification task.

Table 1 presents the results of ablation experiments conducted on various models, including KNN, SVM, MLP, and DGC, aimed at classifying feedback types within the basic view dataset. Notably, we employed embeddings extracted by the fGCL encoder as inputs for the KNN, SVM, and MLP models. The experimental results demonstrate that our model exhibits superior performance in the task of feedback type classification while maintaining a balance between sensitivity and specificity. However, it is worth noting that the absence of the fGCL encoder leads to a noticeable degradation in the performance of the DGC model.

**Table 1:** The entire-period feedback type classification results on the basic view dataset.

**Models**	**ACC**	**AUC**	**F1 Score**	**SEN**	**SPEC**
fGCL + KNN	63.80 ± 0.15%	66.07 ± 0.55%	62.93 ± 0.15%	58.63 ± 0.43%	63.96 ± 0.05%
fGCL + SVM	59.11 ± 0.22%	63.19 ± 0.39%	52.32 ± 0.25%	51.21 ± 0.53%	55.96 ± 0.45%
fGCL + MLP	58.11 ± 0.32%	63.19 ± 0.39%	58.61 ± 0.25%	54.38 ± 0.53%	58.13 ± 0.18%
DGC w/o encoder	61.11 ± 0.12%	63.19 ± 0.39%	58.61 ± 0.25%	57.00 ± 0.53%	64.96 ± 0.45%
**fGCL + DGC (ours)**	**65.12** **±0.45%**	**69.79** **±0.35%**	**64.90** **±0.06%**	**64.71** **±0.35%**	**65.53** **±0.05%**

#### 4.3.1 Individual-level classification

We further examined our hypothesis that whether the emotional contagion occur over the period of sustained interpersonal interaction. To this end, we divided the trial sequence into three stages averagely: early stage, middle stage and late stage to (0 ~ 1/3), (1/3 ~ 2/3) and (2/3 ~ 1) respectively and evaluate them separately by our model. The result in Table 2 revealed that the classification performance of DMT actors in DMT-PST dyad is better than DMT partners in DMT-PST dyad in Table 3 entirely, and the accuracy and the F1 score of DMT actors in DMT-PST dyad represents an evident increasing trend especially from the early stage to the middle stage, and the late stage was superior to the other stages. In contrast, that of DMT partners in DMT-PST dyad displayed a relatively less increment in the accuracy and F1-score. This may additionally reflect that the emotional contagion evolve cumulatively over the sustained interpersonal communication, this, in turn, affects the neurological representation when encountering negative event, and thus resulting in the poor classification performance of recognizing the pattern of neural response of DMT partners in DMT-PST dyad. On the contrary, DMT actors in DMT-PST dyad release negative emotions by transmitting them to their partners, this then gradually break the disordered response pattern resulting in the improvement of classification performance.

**Table 2:** The individual-level classification results of DMT in DMT-PST dyad across early, middle, and late stages with entire period on the basic view dataset.

**Periods**	**ACC**	**AUC**	**F1 Score**	**SEN**	**SPEC**
Entire	68.28 ± 0.05%	70.49 ± 0.05%	70.50 ± 0.06%	66.67 ± 0.08%	67.63 ± 0.20%
Early	60.66 ± 0.14%	64.13 ± 0.06%	64.21 ± 0.17%	58.85 ± 0.29%	65.62 ± 0.25%
Middle	69.33 ± 0.25%	64.35 ± 0.27%	68.23 ± 0.31%	64.51 ± 0.35%	64.52 ± 0.34%
Late	71.33 ± 0.17%	73.31 ± 0.06%	72.64 ± 0.22%	72.83 ± 0.28%	67.50 ± 0.31%

**Table 3:** The individual-level classification results of PST in DMT-PST dyad across early, middle, and late stages with entire period on the basic view dataset.

**Periods**	**ACC**	**AUC**	**F1 Score**	**SEN**	**SPEC**
Entire	63.33 ± 0.05%	67.87 ± 0.06%	67.20 ± 0.08%	66.67 ± 0.10%	63.01 ± 0.13%
Early	59.38 ± 0.25%	62.12 ± 0.13%	64.96 ± 0.35%	60.00 ± 0.46%	67.14 ± 1.17%
Middle	64.21 ± 0.15%	63.19 ± 01.39%	68.59 ± 0.26%	68.20± 0.37%	64.96 ± 01.45%
Late	64.76 ± 0.12%	68.16 ± 0.08%	65.18 ± 0.14%	67.70 ± 0.18%	65.12 ± 0.21%

Specifically, our classifier was evaluated using leave-one-dyad-out cross-validation, and the results of DMT-PST dyad and PST-PST dyad are shown in Tables 2, 3, respectively, which shed light on our aforementioned hypothesis. Regarding the result of DMT actors inside DMT-PST dyads, our model achieved the classification accuracy as 60.66 ± 0.14% at the early stage of problem-solving; this accuracy then increased at the middle stage to 69.33 ± 0.25% and raised to 71.33 ± 0.17% at the late stage. Conversely, the results for DMT partners within DMT-PST dyads displayed a distinct pattern. The accuracy increased from 59.38 ± 0.25% in the early stage to 64.21 ± 0.15% in the middle stage, with marginal change observed in the late stage, yielding an accuracy of 64.76 ± 0.12%. Analyzing this pattern, it becomes apparent that at the early stage of problem-solving, no substantial difference in brain activity existed between DMT actors and DMT partners, indicating that the emotional contagion effect was relatively dormant. As the problem-solving process advanced to the middle stage, the emotional contagion effect commenced within DMT-PST pairs, with DMT actors gradually transmitting the negative emotions stemming from pressure to DMT partners. As a result, the classifier exhibits a significant increase in its ability to differentiate the neural responses of DMT actors when facing different types of feedback. In the final stage of problem-solving, the emotional contagion effect within DMT-PST pairs subsided, with DMT actors transferring their negative emotions to DMT partners. Consequently, the classifier's classification performance on DMT actors continues to increase over time, while there is no such pronounced trend observed on DMT partners. In addition, the results of PST-PST dyads in Tables 4, 5 display no significant changes in the accuracy; this might suggest that the emotional contagion does not appear in PST-PST dyads and thus the neural pattern differentiation in all stages is vague.

**Table 4:** The individual-level classification results of PST1 in PST-PST dyad across early, middle, and late stages with entire period on the basic view dataset.

**Periods**	**ACC**	**AUC**	**F1 Score**	**SEN**	**SPEC**
Entire	75.03 ± 0.01%	85.54 ± 0.09%	82.48 ± 0.01%	72.06 ± 0.01%	68.09 ± 0.05%
Early	76.67 ± 0.05%	80.62 ± 0.25%	79.17 ± 0.15%	67.31 ± 0.03%	63.96 ± 0.05%
Middle	75.11 ± 03.12%	84.19 ± 01.39%	84.35 ± 0.13%	75.61 ± 0.25%	74.96 ± 0.45%
Late	74.12 ± 01.45%	83.79 ± 0.35%	83.21 ± 0.11%	64.71 ± 0.35%	63.53 ± 0.05%

**Table 5:** The individual-level classification results of PST2 in PST-PST dyad across early, middle, and late stages with entire period on the basic view dataset.

**Periods**	**ACC**	**AUC**	**F1 Score**	**SEN**	**SPEC**
Entire	59.83 ± 0.03%	66.38 ± 0.03%	69.13 ± 0.02%	58.46 ± 0.05%	63.96 ± 0.05%
Early	57.17 ± 0.31%	61.69 ± 0.21%	63.37 ± 0.22%	56.19 ± 0.43%	62.96 ± 0.05%
Middle	56.31 ± 0.19%	60.12 ± 0.14%	59.37 ± 0.13%	53.19 ± 0.21%	55.36 ± 0.12%
Late	55.97 ± 0.13%	59.12 ± 0.13%	58.13 ± 0.12%	55.39 ± 0.13%	51.96 ± 0.03%

Overall, the findings suggest that DMT partners appear to play a role in buffering the negative emotions of DMT actors, particularly when considering the progressive evolution of the tasks. Conversely, individuals in the PST-PST dyads experiencing SBS-neutral contexts show no significant difference in their processing of feedbacks. In addition, DMT actors and those in the PST-PST dyad were processing performance feedback in a similar manner especially in the middle and late stages of the tasks. These outcomes lend support to the idea that SBS contagion unfolds progressively over time.

## 5 Discussion

Overall, our findings suggest SBS-based emotional contagion can occur within female dyads in problem-solving contexts and has different consequences on performance for each member of the dyad. While working together on a math task, DMT partners performed worse over time, whereas DMT actors (i.e., those under SBS) performed better and comparable to dyads working in SBS-neutral contexts. Importantly, DMT partners showed evidence of “catching” this initial stress response from the threatened actor. This, in turn, had direct ramifications for DMT partners' performance. In contrast, these relationships were not evident among PST dyad members interacting in SBS-neutral contexts.

### 5.1 Inference of performance decrement in dyadic interaction

Results provide further insight into the dynamic relationship between two individuals performing in domains where their common identity is devalued. Although it seems conceivable that the performance of both DMT actors and partners would suffer when solving problems together in a negatively stereotyped domain, the results provide further support for the notion that non-threatened partners help buffer initially threatened actors from the deleterious consequences of SBS over time, at their own expense. These findings are consistent with past studies showing that the presence of a female role model or competent female partner alleviates performance decrements otherwise typically evident in stereotype threatening contexts (Marx and Roman, 2002; McIntyre et al., 2005; Thorson et al., 2019). Results expand upon this study in several ways. Most notably, by demonstrating that the transference of an individual's stress response onto their partners may be one important factor in buffering women from SBS during dyadic problem-solving interactions, particularly during initial stages of the interaction. Conversely, like past studies demonstrating that increased emotional processing of feedback in SBS contexts has a negative impact on individuals' performance when alone (Forbes et al., 2015, 2018), findings from this study demonstrate that this effect extends to partners in a dyadic interaction, providing a potential mechanism for underperformance effects among these individuals in group problem-solving contexts moving forward. These results not only contribute to our understanding of stereotype threat and its impact but also underscore the complex interplay of emotions, support, and performance within dyadic interactions.

### 5.2 Efficiency in timescale and implications for contagion hypotheses

Present results provide novel insight into how contagion manifests on the order of milliseconds, a much more rapid timescale than previously assumed, to affect performance accordingly using neuroscience methodologies. Moreover, the design of the present study provides a novel yet realistic platform to examine emotion contagion phenomena via EEG or fMRI methodology in future studies. By using iPads, it was possible to capture simultaneous EEG activity in a controlled manner while still allowing participants to have real-time face-to-face interaction. This design also provides implications for contagion hypotheses specific to the mimicry and proximity literature. Because participants only communicated through an iPad webcam, participants were only able to view their partner's face and hear their voice through the webcam during the interaction. This suggests that vocal patterns and facial expressions may have played an integral role in facilitating contagion effects (Hatfield et al., 1993; Neumann and Strack, 2000). Findings provide a more nuanced understanding of the contagion process while also providing a better understanding of a heretofore largely unexamined question in the literature: how social identity threats and SBS manifest in dyadic interactions to have paradoxical effects on performance. More importantly, findings provide further insight into the many ways the gender gap in STEM domains can be perpetuated but also one day nullified.

### 5.3 Graph-based approach for subject invariant emotional representation extraction

Graph structures naturally align with the brain's topology, allowing for the effective modeling of anatomical regions of interest while preserving functional connectivity (FC) information. The proposed fGCL model harnesses semantically meaningful information, i.e., graphs corresponding to the same mathematical problem, to construct both positive and negative pairs. This innovative approach significantly mitigates inter-subject variability in EEG data and has been evaluated as optimal. The subject-invariant representations extracted from FC graphs are well-aligned within the common embedding space. Furthermore, the fGCL employs a spectral graph network capable of convolving across the entire node set with FC connections, integrating valuable cognitive information (Cohen, 2018).

### 5.4 Limitations and future works

Regarding the limitations of the study, it is important to note that when considering EEG data and the spatial limitations associated with the methodology, conclusions based on precise brain locations should always be interpreted with caution. Results should be replicated and expanded upon in future fMRI studies. However, given the temporal constraints of fMRI methodologies in relation to the findings in this study (i.e., these effects may occur on the order of milliseconds), this approach could also present challenges. Additionally, the proposed fGCL encoder was validated with EEG data of young female students (mean age = 25.18 years) and optimized with semantical auxiliary task. It is well-known that age plays a significant role in emotion processing, and different age ranges may exhibit distinct emotion patterns (Ebner and Fischer, 2014). Therefore, further studies should be conducted to encompass various age ranges for a more generalized encoder model.

## 6 Conclusion

In this study, we addressed a critical issue in previous emotional contagion research: the neglect of subject-level differences. The proposed self-supervised learning approach eliminates individual differences by increasing the attraction of positive pairs and reducing the attraction of negative pairs. This alignment results in neurophysiologically meaningful representations of EEG signals in the embedding space, effectively removing subject-specific information. Based on this foundation, we employ a dynamic graph classification model to analyze the temporal effect of emotional contagion. The results suggest that DMT actors and individuals in PST dyads processed feedback in a similar manner, but distinctions emerged in DMT partners' processing patterns, supporting the idea that SBS contagion unfolds gradually over time. Notably, DMT partners appear to buffer the negative emotions of DMT actors.

## Data availability statement

The raw data supporting the conclusions of this article will be made available by the authors, without undue reservation.

## Ethics statement

The studies involving human participants were reviewed and approved by the IRB (Institutional Review Board) at the University of Delaware.

## Author contributions

JH: Conceptualization, Formal analysis, Methodology, Software, Validation, Visualization, Writing—original draft, Writing—review & editing. RA: Data curation, Writing—original draft. ML: Conceptualization, Project administration, Resources, Writing—original draft. CF: Funding acquisition, Writing—original draft.
